# Analyzing Protein Architectures and Protein-Ligand Complexes by Integrative Structural Mass Spectrometry

**DOI:** 10.3791/57966

**Published:** 2018-10-15

**Authors:** Zainab Ahdash, Andy M. Lau, Chloe Martens, Argyris Politis

**Affiliations:** ^1^Department of Chemistry, King's College London

**Keywords:** Chemistry, Issue 140, Mass spectrometry (MS), native MS, ion-mobility, protein complexes, non-covalent interactions, nucleotide binding, DNA binding, molecular dynamics, modelling.

## Abstract

Proteins are an important class of biological macromolecules that play many key roles in cellular functions including gene expression, catalyzing metabolic reactions, DNA repair and replication. Therefore, a detailed understanding of these processes provides critical information on how cells function. Integrative structural MS methods offer structural and dynamical information on protein complex assembly, complex connectivity, subunit stoichiometry, protein oligomerization and ligand binding. Recent advances in integrative structural MS have allowed for the characterization of challenging biological systems including large DNA binding proteins and membrane proteins. This protocol describes how to integrate diverse MS data such as native MS and ion mobility-mass spectrometry (IM-MS) with molecular dynamics simulations to gain insights into a helicase-nuclease DNA repair protein complex. The resulting approach provides a framework for detailed studies of ligand binding to other protein complexes involved in important biological processes.

**Figure:**
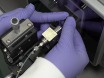


## Introduction

Native mass spectrometric analysis of intact proteins and their complexes is carried out using electrospray and nano-electrospray ionization (nESI), which preserve protein folding and non-covalent interactions during the ionization process^1^,^2^. In native MS, the structure of proteins and their complexes are retained in a near-native state in the gas-phase^3^,^4^. Native MS detects multiple charged protein ions, which are separated according to their mass to charge ratio (m/z) allowing the mass of the protein or protein-ligand complex to be calculated. This information enables the determination of an intact protein's stoichiometry, subunit composition, ligand binding, and interaction networks^3^,^4^,^5^,^6^. Native MS has several advantages compared to other techniques such X-ray crystallography and nuclear magnetic resonance spectroscopy^5^. Firstly, native MS is a rapid and highly sensitive technique, requiring only a few microliters (2-3 µL) of sample at relatively low final complex concentrations in the high nM to low µM range^6^. Secondly, native MS can be used to interrogate heterogeneous protein samples making it possible to analyze multiple proteins and oligomeric states simultaneously. Thirdly, native MS does not require protein samples to be modified before analysis by chemical crosslinking or protein labelling. These advantages have made structural MS a powerful tool for the structural investigation of protein complexes.

Native MS can be combined with ion mobility (IM), a technique that measures the time a protein ion takes to travel through an electric field, enabling the collisional cross section (CCS) to be determined. The CCS provides low-resolution structural information, which enables topology and conformational heterogeneity information of proteins to be obtained. Furthermore, it allows the examination of protein structural models generated by computational approaches.

Protein gas-phase stability can be investigated using collision induced unfolding (CIU) measured by IM-MS. During the CIU process, protein ions are accelerated and activated through increased accelerating collisions with an inert buffer gas within a mass spectrometer^7^,^8^,^9^. This collisional activation process causes the protein to partially unfold, which translates into an increase in CCS. This change in CCS and the energy required to unfold the protein can be measured by IM-MS. Using this approach, the effect of ligand binding on protein stability can be measured^10^. Subcomplexes can be generated in solution using in-solution disruption methods such as the addition of organic solvents to monitor native-like topologies of protein complexes. Disruption of the protein complexes is mainly due to the disruption of intra non-covalent interactions. The sub-complexes maintain native-like topologies and, upon MS detection, reveal information about inter-subunit connectivity.

Integrative approaches in structural biology combine diverse methods to study the structure and dynamics of proteins and their complexes^3^,^4^,^5^,^6^. Native MS and IM-MS have been used to uncover the molecular details of challenging biological systems. There have been several examples of applications including the study of protein assembly pathways^11^,^12^,^13^,^14^, studying protein-protein interaction networks^15^,^16^,^17^, membrane proteins^6^,^18^,^19^,^20^,^21^, and protein-ligand interactions such as nucleic acids^22^,^23^,^24^.

However, native MS also has its limitations. Native MS measurements are often performed in volatile buffers such as aqueous ammonium acetate in which some proteins will not retain their folded native state^3^,^25^. Nevertheless, recent work has shown that this limitation can be overcome by optimization of spraying needle tip diameter (0.5 mm tips) such that protein and protein complex ions can be formed directly from non-volatile buffers with high-ionic-strength that better mimic the physiological environment^26^. Additionally, native MS uses electrospray to ionize and transfer non-covalent assemblies from solution to the gas phase; therefore, the relative abundance of detected complexes may not wholly represent that in solution^5^,^27^. Moreover, in comparison to in solution, the gas phase hydrophobic interactions become weaker and electrostatic interactions become stronger and hence favored^3^,^28^.

In this article, we provide protocols, data analysis, and interpretation for protein identification and ligand binding using native MS, IM-MS, CIU, in-solution disruption, and modelling. The DNA repair complex, HerA-NurA, is used as a model system. DNA double-stranded breaks (DSBs) are one of the most cytotoxic and deleterious forms of DNA damage, resulting in genetic instability and the eventual development of cancer in humans. Homologous recombination is the repair mechanism which eradicates DSBs, a process which is orchestrated by the ATP dependent helicase-nuclease complex, HerA-NurA^22^.

Combining native MS and IM-MS with functional assays and modelling allowed the investigation of: i) the role of NurA in the assembly, conformation, and stability of the complex, ii) the interaction between dsDNA and the complex and its influence on the overall stability of the complex, and iii) the stoichiometry and impact of ATP binding on the assembly^22^. Overall, this work led to an improved understanding of the molecular basis of the HerA-NurA complex by linking protein complex conformational changes and stability with nucleotide binding. This protocol is generic for any protein complex(es) which interacts with one or several ligand(s) types.

## Protocol

### 1. Sample Preparation for Native MS of Protein and Protein-Ligand Complexes

NOTE: To gain an understanding of the molecular basis of a protein complex and ligand binding using native MS, suitable sample preparation is key. The aim of this section is to highlight the essential sample preparation steps prior to MS analysis using the HerA-NurA complex which binds DNA and nucleotides as an example.

1. Prepare 20 µL aliquots of concentrated purified protein (typically 15 - 30 µM) in a 1.5 mL tube.
2. **For ATP or ADP binding analysis** NOTE: Add increasing concentrations of the non-hydrolyzable ATP analog adenosine 5′-*O*-(3-thiotriphosphate), tetralithium salt (ATP-γ-S) or adenosine 5′-diphosphate (ADP). Non-hydrolyzable ATP derivatives generate a stable complex which would enable the ATP-bound protein to be captured. Other non-hydrolysable ATP analogues that could be tested include AMP-PNP and ATP-γ-S-Mg^2+^.
  1. For HerA-NurA studies, mix 5 µM of purified protein with ATP-γ-S and ADP at concentrations ranging from 0-1 mM.
  2. To capture simultaneous ATP-γ-S and ADP binding, add both nucleotides at the same or varying concentrations.
  3. Add 2 mM MgCl_2_ and incubate at 25 °C in a dry bath incubator for 1 h. NOTE: Analysis of nucleotide binding using nESI native MS can result in artefactual binding at high concentrations, therefore non-specific binding must be taken into account^29^. To investigate non-specific binding, add a higher concentration of nucleotides between 2-5 mM).
3. **For DNA binding analysis**
  1. Mix the protein and DNA at a molar ratio that allows for protein-DNA complex formation. For HerA and HerA-NurA, mix 5 µM of purified protein with DNA at a 1:1 ratio.
  2. Incubate the HerA-NurA or HerA - DNA mixture for 30 min at 25 °C in a dry bath incubator until it reaches equilibrium. The duration and temperature of incubation may vary depending on the protein under investigation.
  3. Buffer exchange protein samples to MS compatible buffers. Commonly, aqueous ammonium acetate solution between 5 mM-1 M at pH 7-8 are used. Other MS compatible buffers include ethylenediammonium diacetate (EDDA) and Triethylammonium acetate (TEAA)^30^. For HerA-NurA studies, use 200 mM ammonium acetate pH 7. NOTE: There are several methods for buffer exchange prior to analysis by MS such as using a spin concentrator or chromatography columns. Native MS is mostly limited by the quality of the sample such as buffers and adducts used during purification. Therefore, it is essential to perform sufficient desalting to obtain resolved peaks.
  4. For HerA-NurA ligand binding studies, buffer exchange samples 6-8 times into 200 mM ammonium acetate using a concentrator.Although this method is more time consuming, it ensures that resolved peaks are achieved and allows for accurate mass determination of ATP/ADP bound species.

### 2. Native MS Acquisition and Analysis for Investigating Protein Complexes and Protein-Ligand Complexes

Note: MS conditions should be optimized to achieve highly resolved peaks to enable accurate mass measurements. This section details optimized parameters on a Q-ToF mass spectrometer with a 32k upper limit m/z quadrupole.

1. Prepare in-house capillaries for nano-electrospray and perform instrument mass calibration for accurate mass measurements as detailed by Kirshenbaum* et al.*^1^.
2. Select sensitivity, positive ion acquisition and mobility TOF modes.
3. Turn on the Trap, API and IMS gases. For IM separation, use nitrogen (60 mL/min) and argon (8.4 mL/min for the trap region) as starting points and then adjust.
4. Set an appropriate m/z acquisition range. For an unknown protein, initial optimization steps should use a wide range such as 500-32,000 m/z.
5. Load 2-3 µL of the protein complex solution to be analyzed into a gold coated capillary and insert it into a capillary holder.
6. Gently tighten the capillary and place the capillary in the electrospray source stage and slide the stage into position to start acquiring data.
7. Apply low nano-flow gas pressure (0.00-0.05 Bar) until a drop is formed at the tip of the capillary. The nano-flow pressure can then be dropped until the spray is maintained.
8. Adjust the capillary with respect to the cone by moving the capillary in x, y, z positions and monitor the ion current to achieve a stable ion current. Apply a capillary voltage in the range of 0.9-1.6 kV.
9. Set the sampling cone (50-120 V), source offset (60.0), source temperature (25 °C) and cone gas flow (0.0 L/h). These suggested initial conditions can be adjusted.
10. To acquire a well resolved mass spectrum and to maximize ion transmission, adjust MS parameters and monitor the resulting change in the spectra. These include adjusting the gas flow in the Trap (2-8 mL/min), He Cell (180 mL/min) and IMS cell (90 mL/min) to achieve best separation at maximum transmission.
11. Adjust the trap collision energies if voltage offsets are insufficient. An optimal starting point is between 10-50 V. NOTE: Increasing the trap energy can remove non-covalently bound adducts. However, take care to avoid collision induced dissociation and unfolding of the protein-ligand complex. Perform ion mobility measurements to check if instrument conditions retain the protein in the native folded state (Step 3).
12. Improve desolvation by optimizing the trap bias voltage. An optimal starting point is 20-45 V.
13. Optimize the wave velocity and wave height to achieve best mobility separation. A detailed explanation and protocol can be found here^31^. For the HerA-NurA studies, use wave velocity of 40 (m/s) and wave height of 550-650 (V).
14. Use all other parameters as instrument default values.
15. Prepare a ligand-free sample for analysis as a control for each run (Figure 1). For ligand binding experiments, perform at least three independent measurements.
16. Use the Masslynx software to measure masses of generated species and identify the ligand binding, such as ATP and ADP binding and oligomeric states (Figure 2 **and 3**).Other software available include UniDec^32^, PULSAR^33^ and Amphitrite^34^.
17. To quantify the relative abundance of species, use the corresponding ion intensities observed in the raw ESI-MS spectra (for example ligand bound, different oligomers, *etc*.). Alternatively, perform quantification using specialized software like UniDec and Massign^35^ (Figure 1 **and 3** ).

### 3. Acquiring and Analyzing IM-MS

NOTE: IM-MS separates ions in the gas-phase based on their size (mass), shape and charge. Every feature resolved in m/z spectrum is associated with a drift time distribution. IM-MS measures the drift-time of an ion which can be used to calculate the collision cross section (CCS). Drift time values measured from IM-MS data acquired using a drift-tube can be linearly correlated to CCS values^36^. For travelling wave IM-MS (TWIMS) measurements, calculating CCS values requires a calibration curve obtained from protein standards with known CCS values^37^.Compact structures travel faster than extended or elongated structures due to reduced interactions with buffer gas in the mobility cell^38^. Therefore, IM-MS can be used to detect if the native folded structure has been retained in the gas phase^39^,^40^. This section outlines how to measure IM-MS and calculate the CCS of protein using TWIMS.

1. After optimizing instrument conditions for stable transmission (**Step 2**), reduce the collisional energy and sampling cone as low as possible whilst retaining good spectra quality.
2. Use the optimized wave velocity and wave height to acquire IM-MS (**Step 2**).
3. Measure the ion drift time with IM-MS at three different wave velocities (*e.g., *550, 600 and 650 m/s) whilst maintaining the same wave height (*e.g., *40 V).
4. To determine the protein ions CCS, measure protein calibrants under the same instrument conditions used for protein under investigation. Optimal drift-time calibration requires measurement of proteins with known CCS.
  1. Select four calibrants, two with a mass above and two with a mass below that of the protein under investigation^37^. Most importantly, make sure that the wave height and wave velocity are the same as those recorded for the protein under investigation.
5. Calculate CCS manually^41^ or using a specialized software such as PULSAR^33^ and Amphitrite^34^ (Figure 5).
6. To check whether the protein is native-like in the gas-phase, compare experimental CCS to theoretical CCS obtained from high resolution structures. For HerA-NurA, calculate theoretical CCS using the Projection Approximation (PA) method used in MOBCAL^42^. Other methods include trajectory method (TM)^42^ and exact hard sphere scattering (EHS)^43^.

### 4. In-Solution Disruption of Protein Complexes for Native MS and IM-MS Led Structure Determination

Note: Protein sub-complexes can in some cases be identified from the same solution as the intact complex. However, further structural information such as inter-subunit connectivity and complex assembly can be attained from disrupting protein interactions in solution, to form sub-complexes. This can be achieved in several ways such as the addition of organic solvent, increasing the ionic strength or manipulating the pH. To gain insight into the HerA-NurA complex subunit connectivity and complex assembly, sub-complexes were generated in solution by adding solvents which perturb subunit interactions.

1. Prepare the protein sample and buffer exchange into ammonium acetate as described in **Step 1**.
2. Add 10-40% of solvent in 10% increments. Solvents typically used are Methanol (MeOH), dimethyl sulfoxide (DMSO) or acetonitrile (ACN). NOTE: This can be performed within a polypropylene microcentrifuge tube.
3. Incubate the mixture on ice for 1 h.
4. Acquire an IM-MS spectrum for each condition (**Steps 2 and 3**) (Figure 4).
5. Use the SUMMIT software^44^ to assign protein sub-complexes and generate protein interaction networks. Alternatively, manually generate a list of theoretical masses of the expected species.
6. To ensure subcomplexes are folded, calculate the experimental CCS values for the sub-complexes and compare to theoretical CCS as explained in **Step 3** (**Table 1**, Figure 5 and Figure 6).

### 5. Investigating Protein Complex Stability using Collision Induced Unfolding (CIU)

Note: CIU can be used probe the structural stability of proteins and their complexes upon ligand binding. Specialist software packages such as PULSAR^33^, Amphitrite^34^ and CIU suite^9^ can then be used to model the gas-phase unfolding of the protein under investigation with and without ligand. As an example, this section outlines procedure for monitoring gas-phase unfolding trajectories and investigating the stabilizing effect of DNA and ATP binding on the HerA-NurA complex.

1. Record IM-MS data whilst increasing the trap acceleration voltage from 10 V to 200 V in 2-10 V increments to progressively unfold the protein in the gas-phase. NOTE: Recording smaller increments results in more data files to process, however this approach provides more resolved unfolding plot, which is important for analyzing the transition points between folded/unfolded species.
2. Analyze the data acquired using PULSAR^33^, Amphitrite^34^ or CIU suite^9^ and generate two-dimensional unfolding plots in units of CCS as a function of accelerating voltage (**Step 3**). For each charge state, this is created by stacking the intensity- normalized CCS distributions at each accelerating voltage (Figure 7 **A-Bi**).
3. Generate a theoretical unfolding plot using one of the software packages. The data will be fitted to an unfolding model. This makes it possible to quantify the collisional energy at which unfolding transitions occur and determine the stability of proteins with and without bound ligands^33^. An unfolding transition is when a species transitions from one state (based on their experimental CCS values) to another state with a larger CCS.
4. To quantitate the transitions, calculate the transitional midpoint(s) between states using algorithms and software such as PULSAR^33^. This is commonly reported as CV_50_, which is the collision (trap) voltage value at which 50% of a specific state is depleted.
5. Using the CV_50_ value, calculate the total internal energy of an ion using the center-of-mass collision energy (KE_COM_)^45^. KE_COM_ is defined by the total internal energy available for the unfolding transition of an ion and is calculated from the kinetic energy and masses of the collision partners (protein ion and neutral gas) as described in equation (1)^10^ KEcom (eV) = 
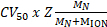
 (equation 1). Where* Z* is the ion charge, M_N_ is the mass of the neutral gas and M_ION_ is the mass of the protein ion. NOTE: This is because CIU of proteins is charge dependent46,47. It is recommended to perform the KE_COM_ analysis to more than one charge state ( Figure 7**Aii**).

### 6. Modelling Procedures for Differential Molecular Dynamics Simulations used in Integrative MS

NOTE: Using models of protein subunits or complexes such as from crystal structures, differential MD simulations (protein complex with and without ligand) can be used to determine effects of for example ligand presence on protein structure and dynamics. This section details a workflow and tools needed for modelling procedures necessary to set-up differential molecular dynamics simulations.

1. Identify the subunits which compose the complex (Figure 8**A**, **in Steps 2 and 3**). Source existing models of subunits, *e.g., *crystal structures from the RCSB databank (https://www.rcsb.org). The UniProt entry of the protein will contain a list of know crystallographic/NMR structures (http://www.uniprot.org). If these are not available, the theoretical sequence can be input to BLAST to identify suitable templates for homology modelling (http://blast.ncbi.nlm.nih.gov/).
2. Assemble the complex in the correct topology (Figure 8**A-ii**). This can be done through various methods. The individual subunits can be fitted into available electron microscopy maps found on the EMDB to assemble the intact complex (https://www.ebi.ac.uk/pdbe/emdb/). A tutorial for fitting PDBs into EM maps using Molecular Dynamics Flexible Fitting (MDFF) can be found here: http://www.ks.uiuc.edu/Training/Tutorials/science/mdff/tutorial_mdff-html/.
3. Identify missing regions of the complex (Figure 8**A-iii**). Perform multiple sequence alignment (MSA) between the PDB and theoretical sequence to identify residues which may be unfitted into crystal structures, or any mutations inherited from crystallographic experiments. MSA can be performed using the webservers such as T-Coffee (http://tcoffee.crg.cat/apps/tcoffee/do:regular).
4. Regenerate missing residues via homology modelling (Figure 8**A-iv**). Missing residues of the protein complex can be built using the MODELLER program (https://salilab.org/modeller/). MODELLER can output an ensemble of n models in different regenerated configurations. Good models can be identified based on their Discrete Optimised Protein Energy (DOPE) score. A comprehensive tutorial is provided on the software website (https://salilab.org/modeller/tutorial/).
5. Perform differential molecular dynamics (MD) simulations of the protein complex (Figure 8**B**) to identify regions of proteins which respond to a particular environmental change, *e.g., *presence of a ligand. In such simulations, behavioural parameters from Simulation A (protein only) which acts as a reference, is subtracted from Simulation B (protein+ligand). The differential root mean square fluctuation (RMSF) calculated between Simulations A and B can inform on regions of the protein which increase or decrease in flexibility in a ligand dependent manner.
  1. Perform MD simulations and downstream analysis using GROMACS (http://www.gromacs.org). A tutorial can be found at: http://www.bevanlab.biochem.vt.edu/Pages/Personal/justin/gmx-tutorials/Lysozyme/index.html. To elimate model bias, the structure of the ligand-bound complex should be generated first. The protein is then copied from this without the ligand, to yield a protein model identical to the ligand-bound complex. 

## Representative Results

Native MS results revealed the oligomeric state, composition and topology of the HerA-NurA complex (Figure 1). As non-covalent interactions are preserved in the gas-phase, native MS of ATP-γ-S and ADP titrations experiments determined the pairwise nucleotide binding to HerA-NurA (Figure 2) and that increasing the ATP-γ-S concentration increases the relative intensity of hexameric HerA (Figure 3). Structural information regarding subunit interactions were obtained from in-solution disruption followed by native MS and were in agreement with and theoretical masses (Figure 4 **and Table 1**).

The experimental CCS values of proteins and their complexes was derived from IM-MS experiments (Figure 5). These values are rotationally averaged gas-phase cross-sectional calculations of the molecular shape, and describe the dimensional state of the protein. CCS values are compared to theoretical measurements from x-ray crystallography and a good agreement infers that the native shape in retained in the gas-phase** (Table 1)**. This validates using CCS values for building low-resolution models of the protein assembly^48^.

Experimental CCSs can be calculated for each charge state ion. A native-like protein conformer may give rise to charge state ions with similar CCS values. However, higher charge state ions increased coulombic repulsions which may lead to protein gas-phase unfolding and larger CCS values compared to the theoretical CCSs. The CCS value of the lowest charge state ions are therefore usually used^49^. For HerA-NurA, in-solution disruption experiments on HerA and HerA-NurA with and without DNA prompted the generation of an assembly pathway starting with monomers then forming the entire hexameric HerA (HerA_6_)-dimeric NurA (NurA_2_) complex with DNA (Figure 6).

Differences in the CIU unfolding plots between the apo (ligand-free) and ligand bound define the change in complex stability upon ligand binding. A higher CV_50 _or KE_COM_ value implies a more stabilized ion in the gas-phase. CIU and KE_COM_ analysis revealed DNA-bound HerA-NurA is more stable than the DNA-free complex (Figure 7**Aii**). From CIU-MS analysis in the respective ATP-binding states, the four-ATP-γ-S bound state reduced complex stability in the gas-phase and the six -ATP-γ-S bound state where are all sites are occupied was the most stable (Figure 7**Bii**). Native MS can reveal the discrete nucleotide binding states of HerA; however, it cannot distinguish which HerA subunits are binding ATP and where this binding takes place. This information can be derived from explicit solvent MD simulations on the hexameric HerA and the HerA-NurA following the summarised Workflow (Figure 8).

**Figure:**
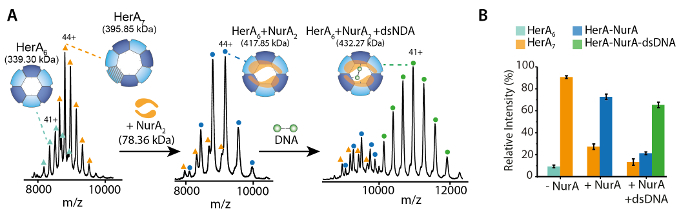

Figure 1. Interrogating the oligomeric state, composition and topology of the HerA-NurA non-covalent complex. (A) Mass spectra of HerA, HerA-NurA and HerA-NurA in the presence of DNA (15.4 kDa 25 bp double- stranded DNA). The HerA sub-complex exists as both a hexamer and a heptamer. NurA dimer binds and to a HerA hexamer imposing oligomeric conversion. The DNA binds the formed HerA- NurA complex (Results adapted from Z. Ahdash* et al.*, 2017^22^). (B) Relative intensities of the identified species are calculated using UniDec^32^. Please click here to view a larger version of this figure.

**Figure:**
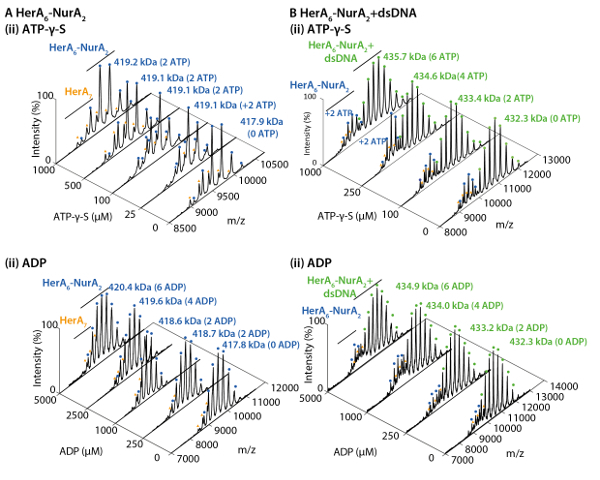

Figure 2. Native ESI-MS reveals the mechanism of nucleotide binding to HerA-NurA. Mass spectra (A) HerA-NurA and (B) HerA-NurA-DNA with increasing concentrations of (i) ATP-γ-S and (ii) ADP. Measured masses are compared to theoretical masses and the amount of ATP-γ-S or ADP bound are determined. Measured masses and number of bound nucleotides are shown on the spectra. ATP-γ-S and ADP titrations experiments determined the pairwise nucleotide binding to HerA-NurA alone and when in complex with DNA indicating a cyclical reaction mechanism (Results adapted from Z. Ahdash* et al.*, 2017^22^). Please click here to view a larger version of this figure.

**Figure:**
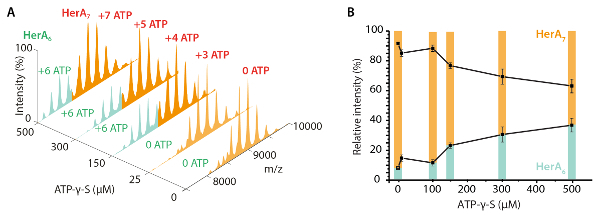

Figure 3. Measuring the effect of increasing ATP-γ-S concentrations on HerA oligomeric state. (A) Mass spectra of HerA at increasing concentrations of ATP-γ-S. (B) Graph showing the relative intensities of different species from native MS calculated using UniDec deconvolution software^32^. As the ATP-γ-S concentration increases, the relative intensity of hexameric HerA also increases. Number of ATP-γ-S molecules bound is shown on the spectra (Results adapted from Z. Ahdash* et al.*, 2017^22^). Please click here to view a larger version of this figure.

**Figure:**
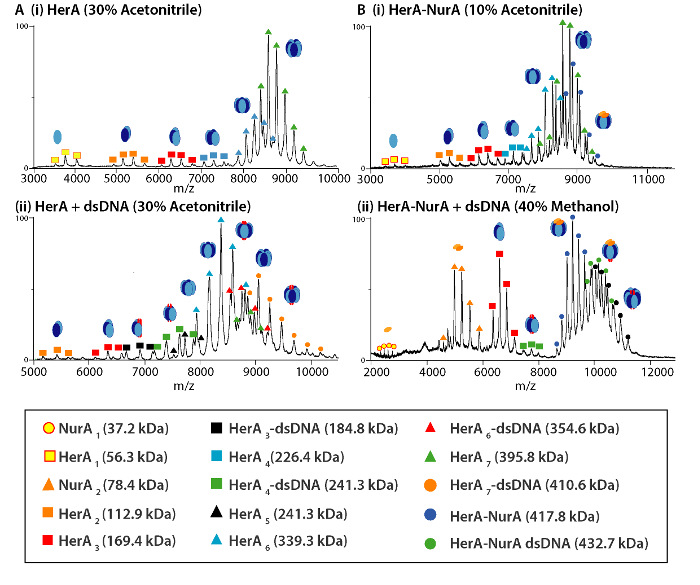

Figure 4. Mass spectra and sub-complex dissociation products of (A) HerA and (B) HerA-NurA (i) alone and in the presence of (ii) DNA following of in-solution disruption. In-solution disruption experiments were performed using 10-40% of Acetonitrile, Methanol (MeOH) or dimethyl sulfoxide (DMSO) and resulted in the formation of various subcomplexes. Please click here to view a larger version of this figure.

**Figure:**
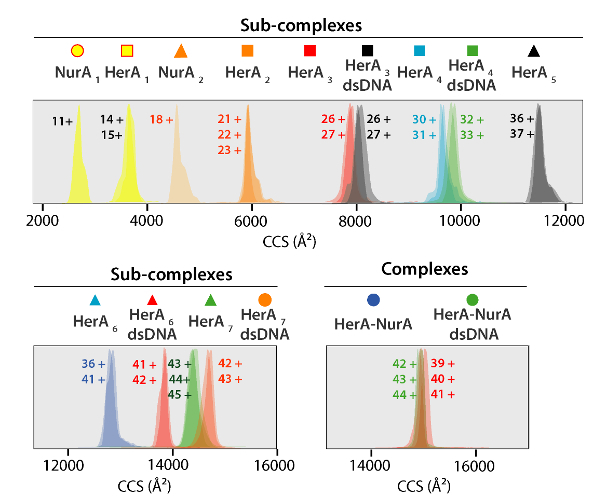

Figure 5. Ion mobility arrival time distributions shown on a CCS axis for complexes and generated sub-complexes. Icon for each sub-complex correlate with those annotated on the spectra in Figure 4. Experimental and calculated masses and CCS values of Sub-complexes are listed in **Table 1** all of which showed an agreement between experimental and calculated values (after considering the typical uncertainty in the resolution of travelling wave ion mobility mass spectrometry of ±5-8%^37^). Please click here to view a larger version of this figure.

**Figure:**
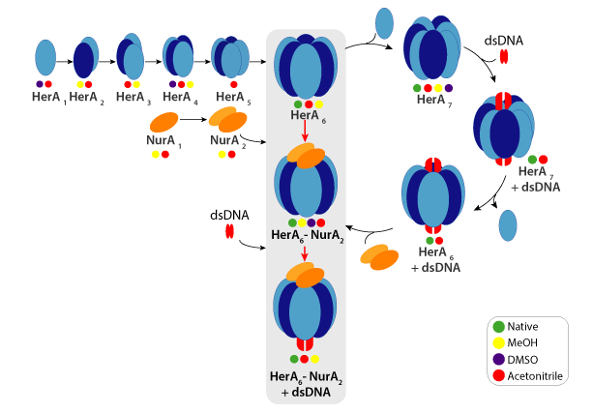

Figure 6. Assembly pathway of the HerA-NurA complex generated from in-solution disruption native MS and IM-MS. Colored circles indicate conditions where each sub-complex was observed: native (green, prior to disruption), Methanol (yellow), DMSO (purple) or Acetonitrile (red). Please click here to view a larger version of this figure.

**Figure:**
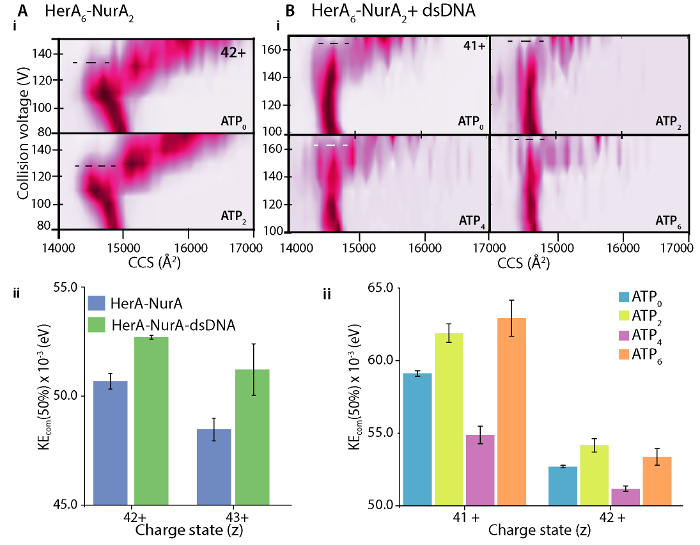

Figure 7. Investigating the stabilizing effect of (A) DNA on HerA-NurA and (B) ATP binding to HerA-NurA-DNA. (i) Gas-phase CIU-MS plots and (ii) center-of-mass collision energies (KEcom) calculation show that that the presence of dsDNA stabilizes the HerA-NurA complex and that the six ATP-γ-S bound state is the most stable. Stabilization for different charge states is shown. Plots were generated using PULSAR 33. Results from (A) adapted from Z. Ahdash* et al.*, 2017^22^. Please click here to view a larger version of this figure.

**Figure:**
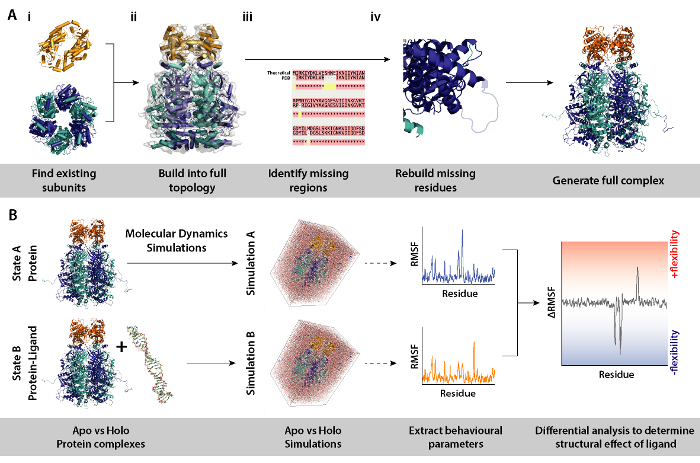

Figure 8. Workflow for the modelling procedures for differential molecular dynamics simulations. (A) Generating the complex under investigation by building the topology from existing subunits and rebuilding missing residues. (B) Workflow for running molecular dynamics simulations on the protein complex with and without ligand. Molecular dynamics simulations are ran for the protein only which acts as a reference and which is subtracted from simulation of protein plus ligand. This is followed by calculating the differential root mean square fluctuation (RMSF) between the simulations and determining the effect of ligand binding. Please click here to view a larger version of this figure.

**Table:** 

**Complex / sub-complex**	**Theoretical Mass (kDa)**	**Experimental Mass (kDa)**	**Theoretical CCS (Å2)**	**Experimental CCS (Å2) [charge]**	**Condition HN**
HerA_6_-NurA_2_	416.22	417.85	14531	14577 [42+] 14599 [43+] 14608 [44+] 14637 [45+]	10-20% ACN, 10-40% DMSO, 10% MeOH
HerA_6_-NurA_2_-dsDNA	431.72	432.27	-	14661 [39+] 14728 [40+] 14781 [41+] 14837 [42+]	10% ACN, 10% MeOH
NurA_1_	39.12	38.18	3254	2618 [10+] 2746 [11+] 2878 [12+]	10-40% ACN, 10% MeOH, 20-40% DMSO
NurA_2_	78.24	78.36	4890	4903 [16+] 4614 [17+] 4537 [18+] 4666 [19+]	10-40% MeOH, 20-40% DMSO
HerA_1_	56.33	56.32	4131	3647 [14+] 3792 [15+] 3950 [16+]	10-40% ACN, 40% DMSO
HerA_2_	112.66	112.95	6475	5648 [20+] 5747 [21+] 5842 [22+] 5996 [23+]	40% Meth, 10-40% ACN
HerA_3_	168.99	169.39	8607	7501 [25+] 7616 [26+] 7717 [27+] 7867 [28+]	10-40% MeOH, 10-40% CAN, 40% DMSO
HerA_3_ + DNA	183.99	184.976	-	7655 [26+] 7990 [27+] 8107 [28+]	10-30% ACN
HerA_4_	225.32	226.2	10477	9205 [30+] 9287 [31] 9493 [32+] 9961 [33+]	10-40% MeOH, 10-40% ACN
HerA_4_ +DNA	240.82	241.33	-	9637 [31+] 9756 [32+] 9830 [33+]	10-30% ACN
HerA_5_	281.65	282.75	11853	10847 [36+] 10958 [37+] 11161 [38+]	30-40% ACN
HerA_6_	337.98	339.3	12517	12335 [38+] 12386 [39+] 12498 [40+] 12590 [41+] 12676 [42+] 13019 [43+]	10-40% MeOH, 10-40% ACN
HerA_6_ +DNA	353.48	354.626	-	12890 [40+] 13081 [41+] 13184 [42+] 13273 [43+] 13463 [44+] 13576 [45+]	30% ACN
HerA_7_	394.3	395.85	13901	14154 [42+] 14219 [43+] 14261 [44+] 14285 [45+] 14335 [46+]	10-40% MeOH, 10-40% ACN, 10-40% DMSO
HerA_7_ +DNA	409.8	410.62	-	14414 [41+] 14510 [42+] 14558 [43+] 14598 [44+] 14630 [45+] 14641 [46+]	10% ACN

**Table 1.** Experimental and calculated masses and CCS values of HerA-NurA and its sub-complexes generated form in-solution disruption studies.

## Discussion

MS is playing an increasingly important role in characterizing the stoichiometry, interactions and subunit architecture of protein complexes. IM-MS data can be used to define topological arrangements of subunits within multicomponent complexes. Compared to other existing structural biology methods, MS has several advantages. Native MS is a rapid and highly sensitive technique and can be used to probe heterogeneous protein samples. When coupled with in-solution disruption experiments, dissociation pathways of protein assemblies can be monitored. Together with crystal structures or homology models, the information offered by structural MS offers a tool for investigating protein-ligand interactions and provide near-native models and assembly pathways^11^.

Here, we describe the necessary experimental procedures for analyzing the stoichiometry and composition of protein-ligand interactions, with one or more ligands, using integrative MS. This includes MS sample preparation, data acquisition, data analysis, and the integration of MS data using computational tools. To do this, we used the DNA-resection HerA-NurA hetero-oligomeric protein complex, bound to three ligands (DNA, ATP, and ADP), as our model system. The protocol shows the use of the currently available software to aid data analysis and presentation.

Acquiring high quality spectra is important for ligand binding analysis, therefore, careful sample preparation steps are critical, including protein purification, ligand titration, and buffer exchange. One limitation of nESI native MS when studying ligand binding is non-specific binding. Non-specific binding occurs during droplet desolvation throughout the electrospray process. This increases the ligand concentrations and therefore alters the protein/Ligand ratio^29^. The binding of nucleotides results in a relatively small mass difference between apo and nucleotide-bound protein which does not alter the ionization efficiency^50^,^51^.

We used the Synapt G2-Si MS system for our work, but the protocols are applicable for different investigations of other protein-ligand complexes using other commercially available nano-electrospray mass spectrometers. Integrative structural MS is increasingly playing an important role in addressing biological problems of greater complexity. The workflow and techniques described here are well-suited for understanding the structural consequences and building mechanisms of protein complex and protein-ligand formation which are otherwise difficult to study using conventional structural techniques.

## Disclosures

No conflicts of interest declared.
